# Use of Artificial Intelligence for Diagnosing Oral Mucosa Conditions: A Review

**DOI:** 10.3390/diagnostics16020365

**Published:** 2026-01-22

**Authors:** Bianka Andrzejczak, Aleksandra Diedul, Anna Szczepankiewicz, Piotr Trojanowski, Antoni Skrzypczak, Anna Bączkiewicz, Hanna Szymańska, Marzena Liliana Wyganowska, Zuzanna Ślebioda

**Affiliations:** 1 Medical Faculty, Poznan University of Medical Sciences, ul. Fredry 10, 61-701 Poznań, Poland91207@student.ump.edu.pl (A.D.);; 2 Department of Periodontology and Oral Mucosa Diseases, Poznan University of Medical Sciences, ul. Bukowska 70, 60-812 Poznań, Poland

**Keywords:** artificial intelligence, oral mucosa, dentistry

## Abstract

Artificial Intelligence (AI) is a computer science that focuses on developing systems and machines capable of performing tasks that typically require human cognitive abilities. It has widespread applications in medical diagnostics. Its use has led to rapid advancements in diagnostic methodology, enabling the analysis of large datasets. The major applications of AI in medical diagnostics include personalized treatment based on patient genetics, preventive measures, and medical image analysis. AI is employed to analyse genomic data and biomarkers, aiding in the precise tailoring of therapies to individual patient needs. It could also be employed in modern dentistry in the near future, helping to achieve higher efficiency and accuracy in diagnosis and treatment planning. AI may be utilized in screening for oral mucosa lesions and to discriminate between oral potentially malignant disorders and cancers from benign lesions. The potential advantages of AI include high speed and accuracy in the diagnostic process, as well as relatively low costs. The aim of this review was to present the potential applications of AI methods in the diagnosis of selected mucocutaneous diseases. A literature review focuses on oral lichen planus, recurrent aphthous stomatitis, and oral and laryngeal leukoplakia.

## 1. Introduction

Artificial Intelligence (AI) is a computer science that focuses on developing systems and machines capable of performing tasks that typically require human cognitive abilities. It encompasses skills such as machine learning, pattern recognition, reasoning, natural language understanding, and decision-making [1,2]. AI has widespread applications in the field of medical diagnostics. Its use has led to rapid advancements in diagnostic methodology, enabling clinicians to make more informed therapeutic decisions [1,2,3]. AI-based programmes enable the analysis of large datasets [3]. Significant applications of AI in medical diagnosis include personalized treatment based on patient genetics, preventive measures, and the analysis of medical images [4]. AI is employed to analyse genomic data and biomarkers, aiding in the precise tailoring of therapies to individual patient needs. Through AI, it is easier to understand which genetic mutations predispose individuals to specific diseases, leading to better personalisation and enhanced treatment efficacy [4]. AI enables the investigation of patient data, including test results, family medical history, lifestyle, and other factors, to assess the risk of certain disorders, such as heart disease, diabetes, stroke, or cancer. Based on these analyses, AI systems can predict which patients are most at risk of developing a particular disease, enabling early preventive interventions [5]. AI is used to analyse images such as X-rays, computed tomography scans, magnetic resonance imaging, and ultrasound. Machine learning algorithms are trained to recognise pathologies in images, such as tumours, neoplastic changes, bone fractures, and other abnormalities. Thanks to AI, systems can detect subtle, hard-to-identify changes [6].

In the development of AI, artificial neural networks (ANNs) play a crucial role. ANNs are mathematical models inspired by the human brain’s functioning, enabling the identification of complex relationships and patterns, as well as decision-making and data analysis. They consist of neurons, which are computational units connected in layers. Each “neuron” processes information using weights and activation functions. They include an input layer responsible for receiving data, hidden layers that process the data, and an output layer that provides the result [7]. Neural networks learn from training data, adjusting the connections between neurons to accurately predict outcomes. For example, by analyzing and comparing images of a human face and a tree, neural networks learn to identify the details that distinguish a human face from a tree. In medicine, neural networks can learn to differentiate preparations showing normal tissues from those showing diseased tissues [7].

Oral diseases, including malignant and premalignant lesions, usually develop on preexisting chronic tissue alterations. They are frequently difficult to discern through conventional visual examination, which heavily relies on the clinician’s expertise and subjective interpretation. Therefore, additional diagnostic measures, including incisional and excisional biopsies, cytological techniques, and optical approaches, are often utilized, with a scalpel biopsy remaining the gold standard for diagnosing potentially malignant lesions, offering a diagnostic accuracy of up to 88.9% [8]. All of those methods are, to some extent, invasive and relatively expensive. In recent years, some cost-effective alternatives, like brush biopsies, tissue autofluorescence, and chemiluminescence (e.g., toluidine blue staining), have been introduced [8]. Nevertheless, there is an urgent need to improve and simplify the diagnostic process for oral mucosa diseases.

The aim of this review was to present the potential applications of artificial intelligence methods in the diagnosis of selected mucocutaneous diseases.

## 2. Materials and Methods

A literature review was conducted on oral lichen planus (OLP), recurrent aphthous stomatitis (RAS), and oral and laryngeal leukoplakia. We revised PubMed, Web of Science, Scopus and Google Scholar from January 2020 to February 2025 using the following keywords: (“Lichen Planus” [Mesh]), (“Lichen Planus, Oral” [Mesh]), (“Artificial Intelligence” [Mesh]) AND (“Lichen Planus” [Mesh] OR “Lichen Planus, Oral” [Mesh]), (“Recurrent aphthous” [Mesh]) AND (“Artificial Intelligence” [Mesh]), (“Leukoplakia” [Mesh]) AND (“Artificial Intelligence” [Mesh]). The inclusion criteria comprised studies addressing selected mucocutaneous disorders (oral lichen planus, recurrent aphthous stomatitis, oral leukoplakia, laryngeal leukoplakia) that examined the application of artificial intelligence in diagnostic procedures. Studies were excluded if they did not directly concern the specified disease entities or if the full text was not available. Duplicates were removed. Ultimately, 16 publications were qualified for the literature review (6 on oral lichen planus, one on recurrent aphthous stomatitis, five on oral leukoplakia, four on laryngeal leukoplakia). Although the primary focus of this review is on oral mucosal diseases, laryngeal leukoplakia was included due to its shared epithelial origin, premalignant potential, and comparable diagnostic challenges. Moreover, artificial intelligence models developed for analyzing mucosal surface lesions are often transferable across anatomical regions of the upper aerodigestive tract, supporting broader clinical applicability. The summary of the included studies is presented in Table 1.

## 3. Results

### 3.1. AI in the Diagnostics of Recurrent Aphthous Stomatitis

Recurrent aphthous stomatitis (RAS) is the most common benign erosive–ulcerative oral condition, affecting up to 20% of the population [20]. Lesions occur singly or multiply and are characterised by recurrence and painfulness. Due to the discomfort caused, such changes can lower patients’ quality of life. Aphthae require differentiation from other oral erosive and ulcerative conditions, including neoplasms [21,22]. The use of AI in RAS is still poorly explored. CNNs were utilized in recognising RAS is the study by Zhou et al. [14]. The study utilized 785 oral photographs of patients from the Department of Stomatology at Zhejiang University Hospital, collected between March 2022 and March 2023. The photographs were divided into three groups: 251 images of patients with RAS, 271 images of healthy mucosa, and 263 images showing other common oral mucosal diseases [14]. All photographs were evaluated by two experienced physicians who recognised the image types and marked the boundaries of disease changes. To perform the tasks, the photograph set was randomly divided into training (628 photos), validation (78 photos), and test (79 photos) sets in an 8:1:1 ratio. The experiments utilized computer hardware equipped with an NVIDIA GeForce 3060 graphics card and PyTorch 1.10, along with CUDA 11.4 software. For image classification, CNN neural networks and three widely used models were employed: DenseNet121, ResNet18, and ResNet50. A non-pre-trained ResNet50 was also included for comparison with the pre-trained ResNet50 model to verify the impact of pre-training. The mentioned models were trained using transfer learning, allowing for their preliminary training on large datasets. The classification process involved automatic extraction of key image features and assignment to one of three classes: RAS, healthy mucosa, or other mucosal diseases [22]. The second stage of the study involved detecting lesions in images to precisely define aphtha boundaries. For this task, YOLOV5 and Faster R-CNN models (pre-trained) were used. A non-pre-trained YOLOV5 model was also used for comparison. The models not only classified images but also predicted the location of disease changes, requiring a loss function considering both classification and location precision [14]. To evaluate model effectiveness, various parameters were used. For classification, precision, sensitivity (recall), F1-score, specificity, and area under the ROC curve (AUC) were considered. For detection, precision, sensitivity, F1-score, and area under the Precision–Recall curve (PR AUC) were analysed. In the image classification task, the pre-trained ResNet50 model proved to be the best, achieving a precision of 92.86%, a sensitivity (recall) of 91.84%, an F1-score of 92.24%, a specificity of 96.41%, and an AUC of 98.95% [14]. The same model, but non-pre-trained, achieved the worst results in all categories. In the object detection task, the pre-trained YOLOV5 model yielded the best results (precision: 98.70%, sensitivity: 79.51%, F1-score: 88.07%, PR AUC: 90.89%) [14]. However, this model, when not pre-trained, had the worst validation accuracy. The study made several key observations. It indicated that pre-trained models were more effective due to transfer learning [14].

Several study limitations were also noted. For example, classification included only 3 categories of changes. It is therefore unclear how much the models’ effectiveness would change if the number of possible categories were expanded. The need for further studies on larger samples was stated, and it was emphasised that despite high effectiveness in recognising mucosal changes by AI models, the presence of a physician in monitoring the diagnostic process, accurately assessing risk, and determining treatment method remains essential [14].

Despite these promising results, the use of computer vision and machine learning in RAS remains an emerging field, with only a limited number of AI-focused studies currently available in the literature. In the object detection task, the YOLOv5 model demonstrated a notable discrepancy between its high precision (98.70%) and lower sensitivity (79.51%). This gap likely arises from the model’s conservative detection threshold; while it is highly accurate at identifying clear lesions without producing false positives, it may fail to detect more subtle, small, or atypical ulcers, leading to a higher rate of false negatives. Consequently, we hypothesize that while YOLOv5 could serve as a highly reliable tool for confirming a diagnosis in clinical practice, its current sensitivity suggests it should be utilized primarily as a supportive screening aid rather than a standalone diagnostic system.

### 3.2. AI-Supported Diagnostics of Oral Lichen Planus

Lichen planus (LP) is a chronic disease with a probable autoimmune basis. It can manifest on the skin as well as mucous membranes, with a very common variant being oral lichen planus (OLP). It shows various clinical forms, including reticular, erosive, atrophic, plaque-like, and bullous [7,23,24]. The etiopathogenesis of lichen planus is complex and not fully explained. It encompasses endogenous factors, including immunological, genetic, and psychogenic triggers, as well as exogenous factors such as medications, infections, or environmental stimuli [7,23,24,25]. During the disease, autoimmune reactions occur, in which T lymphocytes attack keratinocytes, resulting in their damage and the formation of characteristic skin or mucosal lesions [7]. The condition has a chronic and recurrent course, with an associated risk of malignant transformation of approximately 1.37%. Risk factors for this transformation include female sex, lesion location on the tongue, tobacco smoking, alcoholism, and hepatitis C virus (HCV) infection [7,23,24]. Diagnosis of OLP relies on clinical, histopathological, and immunological analysis; however, due to the similarity of lesions to other conditions, such as cheek hyperkeratosis caused by chewing or friction, leukoplakia, lupus erythematosus, or chronic oral inflammation, it can be challenging, especially for general practitioners [7,23,24,25]. For this reason, modern artificial intelligence tools, including convolutional neural networks (CNNs) [7], are increasingly utilized in diagnostics to facilitate more accurate analysis of clinical photographs and the differentiation of OLP from other pathologies [23,24].

In the recent study by Yu et al., the effectiveness of three different artificial intelligence systems (ChatGPT-4O, ChatGPT with Diagram-Date extension, and Claude Opus) was compared in recognizing OLP based on images of mucosal lesions [9]. The study included a total of 128 patients with confirmed OLP, and the assessment was based on the latest International Classification of Diseases ICD-11 standards, which is a key element in evaluating the study’s reliability. Three independent specialists analysed the images of lesions. The AI models were divided into “pre-trained” models, which contained patient case descriptions, and “non-pre-trained” models, which generated results without prior adaptation to this type of diagnosis. Pre-trained models outperformed non-pre-trained ones, achieving the highest diagnostic accuracy in the buccal mucosa (85% vs. 80%) and near-perfect results in detecting gingival changes (100% vs. 90%). The best results were obtained for ChatGPT–Diagram-Date, followed by ChatGPT-4O, with the lowest for Claude Opus. After pre-training, the Chat–Diagrams model showed the highest or near-highest diagnostic accuracy in most analysed locations of oral changes. The largest number of cases involved the buccal mucosa (*n* = 70), where the model correctly identified 59 changes (85%), while Chat-4.O achieved 80% accuracy (56 correct identifications). For the tongue (*n* = 35), differences between models were small—Chat-4.0 correctly identified 25 cases (71%), while Chat–Diagrams identified 24 (69%). Chat–Diagrams achieved the best result in analysing gingival changes (*n* = 10), with 100% accuracy (10/10), compared to 90% (9/10) for Chat-4.0. Although the sample in this last location was small, these results confirmed the high effectiveness of the model after pre-training [9]. The study clearly demonstrated that AI-based methods were most effective in recognising changes on the buccal mucosa, both due to the largest number of cases and the highest detection accuracy, while effectiveness was markedly lower in other oral areas (tongue, gingiva, and other regions) [9].

In the study by Osipowicz et al., data from 80 patients suspected of having OLP were analysed [10]. ANNs were used as an alternative to traditional statistical analysis methods. The final predictive model achieved 85% reliability in the test sample and 71% accuracy in the validation sample, which aimed to verify the system’s effectiveness. The analysis was conducted in two variants: the first, narrower, included only cases where LP was confirmed both clinically and histopathologically; the second variant included cases confirmed clinically with an ambiguous histopathological result. In the studied group of 80 individuals, oral lichen planus (OLP) was histopathologically confirmed in only 4 participants (5.0%). In 57 individuals (71.2%), this diagnosis was not histopathologically confirmed; in 30 (37.5%), it was not unequivocally excluded; and in 31 (38.8%), lichen planus was ruled out. Similarly to previous studies, changes diagnosed as OLP most commonly occurred on the buccal mucosa (93.9%), followed by the gingiva (59.7%), the mucobuccal fold (26.8%), the tongue (26.8%), the palate (7.3%), and the vermilion border (7.3%). Significant predictors included white patches under the tongue and erosions on the mandibular gingiva. The highest diagnostic reliability was observed for the buccal mucosa. The neural network model achieved a classification accuracy of 71%. The study also confirmed that lesion location is not an unequivocal diagnostic criterion [10].

Keser et al. developed an AI model for identifying OLP based on photographs of the buccal mucosa [11]. The study utilized anonymized photographs of 65 healthy mucous membranes and 72 cases of LP, which were analyzed and verified by experts in oral medicine and maxillofacial radiology. The data were divided into training and test sets. The analysis used the Google Inception V3 architecture based on TensorFlow. The results showed that the AI model achieved 100% effectiveness in classifying test photographs for both healthy and diseased mucous membranes. The study results suggest that the use of AI can significantly streamline the diagnosis of OLP.

The study by Achararit et al. involved AI to differentiate OLP from non-OLP changes based on clinical photographs [7]. Three models were used: Xception, ResNet152V2, and EfficientNetB3. The analysis included 609 photographs of OLP and 480 photographs of non-OLP changes, with diagnoses confirmed histopathologically. Fifty-five random photographs from each group were selected for testing, with the remainder used for training. The effectiveness of the CNN models was evaluated using various indicators, including accuracy, positive and negative predictive value, sensitivity, specificity, and F1-score. The best results were achieved by the Xception model, with an accuracy of 88.18% and an F1-score of 88.70%. ResNet152V2 and EfficientNetB3 had slightly lower effectiveness but still allowed for effective diagnosis.

The study by Idrees et al. concerned the development of a machine learning algorithm for analysing inflammatory cells in oral changes using QuPath, version 0.2.3 [12]. To create the model, 24 areas of connective tissue were used from 20 individuals with OLP. The validation set was obtained from a pathology archive and comprised four diagnostic groups: OLP, OLLs (lichenoid changes), OEDLs (changes with epithelial dysplasia), and keratosis without epithelial dysplasia (KWD). The cases without clinical information, unreadable microscopic preparations, or artefacts, and cases that did not fit one of the four diagnostic categories were excluded. The study used 130 H&E preparations, scanned at 40× magnification. To standardise staining, the “estimate stain vector” function in QuPath^®^ software was applied. An oral pathologist selected stromal areas (ROIs) of 0.25 mm^2^ from preparations containing the most inflammatory cells. Cell nuclei segmentation was then performed using “watershed”-based cell detection algorithms. An artificial neural network (ANN-MLP) was applied to classify cells into three types: mononuclear, granulocytes, and others. The “OLP-UWA2021” algorithm was developed to calculate the number of inflammatory cells (mononuclear and granulocytes) in each area. Statistical analysis was conducted using IBM SPSS software, version 27, and diagnostic accuracy was evaluated using the ROC curve. The study involved 130 individuals, of whom 63.8% were women, with a mean patient age of 59.8 years. OLP was most commonly diagnosed (38.5%), followed by OLL (26.9%), OEDL (18.4%), and EHK (16.2%). The most common site of changes was the buccal mucosa, followed by the lateral tongue surface and keratinising mucosa (gingiva and palate). The analysis results showed that the number of inflammatory and mononuclear cells differed significantly across the diagnostic groups. The highest number of inflammatory and mononuclear cells was found in OLP, and the lowest in EHK. Statistical analysis revealed that these differences were significant, except for the comparisons between the OEDL and OLL groups. The machine learning algorithm demonstrated excellent ability to differentiate OLP from other diagnostic groups, achieving AUC values of 0.982 for inflammatory cell count and 0.988 for mononuclear cell count. Proposed diagnostic thresholds for differentiating OLP are 1887 inflammatory cells per 0.25 mm^2^ and 1079 mononuclear cells per 0.25 mm^2^. At these thresholds, OLP diagnosis accuracy was 93.08% using inflammatory cell count and 94.62% using mononuclear cell count. Using the mononuclear cell count was associated with 100% sensitivity. The study developed a machine learning algorithm for diagnosing OLP based on standard H&E-stained preparations. The algorithm utilizes an artificial neural network (ANN) in QuPath software, enabling the objective and efficient evaluation of inflammatory cell counts, including mononuclear cells, in OLP samples. The method is easy to use, requires no additional tissue processing, and eliminates subjectivity. The proposed threshold, based on mononuclear cell count, showed 100% sensitivity and 95% accuracy, although specificity was 91.25%. The study had limitations, including its retrospective nature and the lack of clinical follow-up data; however, it demonstrated that the algorithm is a robust diagnostic tool that requires further validation in multiple centers.

In the study by Qing and Yang, AI enabled precise identification of differences in gene expression between patients with different OLP types, REOLP (recalcitrant erosive oral lichen planus) and SOLP (stable oral lichen planus), allowing for the identification of genes related to inflammatory response, B cell activation, and neutrophils [13]. Using AI algorithms, such as weighted gene co-expression network analysis (WGCNA), key gene groups were identified that were particularly associated with clinical features, including B cell activation and inflammatory response, which are significant for disease development and treatment response. Additionally, the study employed a predictive model based on a neural network, allowing for the prediction of treatment outcomes in OLP patients based on gene expression differences. One hundred most differentiating genes were selected, enabling the creation of a model with high prediction accuracy (80%), which outperformed traditional clinical diagnosis (60%) in forecasting treatment outcomes. The model’s performance was evaluated using a confusion matrix and ROC analysis, allowing for further parameter optimisation. The use of AI in this study enabled a deeper understanding of the molecular basis of OLP and the development of a more precise predictive tool that supports clinical decision-making and contributes to improved patient treatment [13].

### 3.3. AI-Assisted Diagnosis of Oral Leukoplakia

Oral leukoplakia (OL) is a condition characterized by white patches on the mucosa, as defined by the World Health Organization (WHO), which cannot be removed by rubbing and cannot be classified as another condition during diagnostic procedures [26]. Its prevalence varies across studies. Global data indicate an average prevalence of about 2.6%. Early diagnosis and effective treatment of leukoplakia are crucial, as it is classified as a precancerous condition. The rate of malignant transformation of OL ranges from 0.1% to 17.5%. However, OL diagnosis faces several challenges [26]. Standard methods, such as biopsy and histopathological examination or exfoliative cytology, are invasive, and fluorescent devices show low specificity. Additionally, OL is most often detected at an advanced stage, when the risk of malignant transformation is significantly higher than at earlier stages. In the era of artificial intelligence development, ideas for supporting OL diagnosis with AI algorithms are emerging [15].

In the study by Kouketsu et al., the effectiveness of the Single Shot Multibox Detector model was analysed on tongue photographs from 360 patients with oral squamous cell carcinoma (OSCC) and OL, and a control group (64 individuals with other benign tongue changes) [15]. Sensitivity of 93.9% and specificity of 81.2% were achieved for the detection of OSCC, and a combined sensitivity of 83.7% and specificity of 81.2% for both OSCC and OL. However, only the tongue surface was analysed, based exclusively on colour tone changes. Results solely for OL were not included, and the detection sensitivity significantly decreased when they were included. The algorithm also struggled with overexposed photographs, those taken in poor lighting conditions, and detecting small changes, resulting in false-negative results.

A study by Schmidl et al. was based on similar assumptions, but it also included not only photographs but the patient’s clinical history. The utility of ChatGPT-4.0 in diagnosing OSCC and OL was examined [16]. In the first scenario, analysing only photographs, sensitivity of 18.2% and specificity of 52.2% were obtained for OSCC, and sensitivity of 72.2% and specificity of 92.6% for OL. In the scenario including clinical history, sensitivity increased to 100% and specificity to 88.2% for OSCC, and to 93.3% and 96.7% for OL. However, ChatGPT classified photographs without changes as benign changes or OL. It also had difficulties in diagnosing pharyngeal changes, correctly classifying them only when clinical history was added. Thus, the authors investigated the second scenario since in the clinical setting many patients do not show the lesions, but complain about symptoms. In this approach the image was used in prompt, with additional information about the clinical history. In the second scenario, the sensitivity and specificity of ChatGPT increased to 100% and 88.2%. According to Schmidl et al. the pain was most relevant symptom that might have led to the diagnosis of oral/oropharyngeal cancer [16].

An additional approach based solely on clinical history suggested that ChatGPT attaches greater importance to textual description than to images. The number of cases analysed by ChatGPT was only 45, including 15 with OL. The results thus require verification in further studies on a larger patient group.

The evaluation of skin cancer by digital dermoscopy images utilized to support specialists in establishing the diagnosis was an object of several studies. Meanwhile the use of artificial intelligence tools for the diagnosis of oral cavity/oropharyngeal squamous cell carcinoma has not been widely examined so far. As the authors emphasized, most of these approaches were highly technical, using Machine Learning to discriminate oral lesions between normal, premalignant, and tissues with laser-induced autofluorescence spectra recordings or histopathological images [16].

A study on a much larger scale was conducted using 4161 photographs of patients from the Charité—Universitätsmedizin Berlin clinic [5]. The images included healthy mucosa, leukoplakia (LP), lichen planus (OLP), and oral squamous cell carcinoma (OSCC) at various stages. Three thousand three hundred thirty-seven photographs were used for model training, 412 for tests, and 412 healthy photographs as a reference. The Mask R-CNN system with Swin Transformer architecture analyzed photographs pixel by pixel, identifying changes previously manually marked by specialists and classifying them into appropriate disease entities. The highest effectiveness was achieved in detecting OSCC, yielding results of F1 = 0.852 and AUC = 0.974. Unfortunately, LP diagnostics were moderately effective, as the model achieved an F1 score of 0.796 and an AUC of 0.938. These results clearly indicate that LP diagnostics are challenging and often lead to misdiagnosis as OSCC. The model encountered challenges similar to those faced by clinicians. OSCC was most commonly misclassified as oral leukoplakia. The authors attributed this observation to the fact that leukoplakia with dysplasia was included as early-stage OSCC. The misclassification of leukoplakia as OLP was also common in this study that underscores the complexity of differentiation. Although the presented method appears promising, it cannot be widely used in LP diagnostics at present and requires further research and improvement.

AN original approach was presented in a study on mild leukoplakia (without high-grade dysplasia) in 35 patients, using a neural network for classification [17]. Oral photographs taken were converted to a four-bit greyscale, then analysed using appropriate programmes for selected textural features: run-length matrix, co-occurrence matrix, and Haar wavelet transform. The neural network assigned the analysed images to healthy or pathological tissues. The neural network achieved 100% sensitivity and 97% specificity (one case misclassified as pathological). The presented results are promising; however, the method requires further studies on a larger number of patients.

Unlike most studies that focus on analyzing digital photographs of oral changes, the study by Cai et al. focused on analyzing histological preparations to assess genomic changes, particularly the loss of the 9p segment, in oral leukoplakia (OL) and head and neck squamous cell carcinoma (HNSCC) [4]. This approach offers a cheaper and simpler solution compared to traditional genetic diagnostic methods, which detect this mutation, considered one of the earliest biomarkers of oral squamous cell carcinoma (HNSCC) development, especially in cases of leukoplakia. The study analysed 310 OL cases (217 for training, 93 for validation) and 365 HNSCC cases, using deep learning models: Twins-SVT, ResNet50, and Inception_v3 for preliminary data analysis. The highest effectiveness in diagnosing leukoplakia was achieved by the XGBoost model (AUC = 0.890 for the validation set and 0.762 for the test set). However, the model requires further validation, especially in larger, prospective studies, to confirm its effectiveness in various groups [4].

### 3.4. AI for Diagnosing Laryngeal Leukoplakia

Diagnostic systems utilizing AI have also been applied in studies on leukoplakia of the vocal folds and larynx, as well as in the diagnosis of laryngeal neoplasms. That can be considered a complementary extension of AI-based diagnostics of mucosal diseases. Given the morphological similarities, shared premalignant characteristics, and reliance on image-based diagnostic methods, laryngeal leukoplakia represents a relevant model for evaluating the translational potential of artificial intelligence systems developed for oral mucosal pathology.

A system developed for automatic detection and classification of benign tumours based on endoscopic images was described by Kim [6]. It aimed to enable the preliminary screening of vocal fold tumors (cysts, granulomas, leukoplakia, nodules, polyps) and their classification at home. Four CNN models were used: Mask-50, Mask-101, Yolo-4, and SSD-MN to analyse vocal cord endoscopic images. They were tested on a set of 2183 images showing various tumour types: cysts, granulomas, leukoplakia, nodules, and polyps. Yolo-4 obtained the highest F1-score (0.8499), accuracy (0.9395), precision (0.8830), sensitivity (0.8191), and specificity (0.9713), and was thus considered the best model for home pre-screening, both in computer and embedded device versions. The project offers the possibility of transferring the system to embedded and mobile devices, facilitating diagnostic accessibility at home. The application may support physicians in remote diagnostics, reducing waiting time for diagnosis. Drawbacks of the above study include the small number of data (2183 images), which may affect model accuracy, especially for rare tumour types.

Another study on vocal folds involved using an AI system based on multi-instance learning (MIL), which analysed laryngoscopic images and combined results in the context of the entire patient [18]. It was tested on laryngoscopic videos, enabling the evaluation of its ability to diagnose vocal fold leukoplakia (VFL) in real-time. The study aimed to develop an AI system based on multi-instance learning (MIL) to differentiate between benign and malignant VFL. Therefore, participating patients were divided into two groups: malignant (squamous cell carcinoma, carcinoma in situ, severe dysplasia) and benign (inflammation, hyperplasia, mild dysplasia). The developed AI model demonstrated higher accuracy (85%) and AUC (0.851) compared to the group of less experienced physicians (64.8%). Additionally, AI support improved diagnostic results among otolaryngologists. The study had limitations, including the exclusion of normal VFL cases and the use of only white light in laryngoscopy [18].

In a study by Wei et al., an attempt was made to create a laryngoscopic image classification model using deep machine learning to support the diagnosis of laryngeal diseases (cancer, polyps, vocal fold nodules, or cysts), allowing the device to automatically recognize these diseases based on laryngoscopic images [3]. Local features (ResNet) and global features (Transformer) were integrated for comprehensive image analysis. The dataset (Laryngoscope8) contained pathological and unchanged images. To prepare them, the YOLOv5 network was used to extract regions of interest (ROI), i.e., key laryngeal areas. It achieved higher accuracy and AUC, indicating better effectiveness in classifying eight types of laryngeal pathological changes. The NDF-Net-ROI model yielded better results, achieving 2.46% higher accuracy compared to the model analyzing entire images, as unnecessary information was eliminated and the focus was on essential image features. Noted limitations of this project include photographs from various devices, which could affect their quality, and uneven sample numbers in the dataset, which may lead to difficulties in classifying certain diseases (e.g., laryngeal cancer, cysts). However, using ROI, which eliminated irrelevant information, effectively improved results [3].

In another study, elastic scattering spectroscopy (ESS) was used to differentiate between patients with laryngeal cancer and those with laryngeal leukoplakia [19]. During this experiment, ESS effectiveness was evaluated in the context of anatomical locations within the oral cavity, as well as in differentiating smokers from non-smokers. 1086 ESS spectra from various oral sites were analysed. The best results were obtained for the lateral tongue surface (AUC 0.77, sensitivity 0.97, specificity 0.47) and in the group of former smokers (AUC 0.73 vs. 0.63 in current smokers). This study had drawbacks such as a small number of participants (only 20 individuals) and a lack of consideration for other significant factors (alcohol consumption) that could influence the development of pathological changes. Advantages include the non-invasiveness of this method, which may in the future serve as a screening and detection tool for laryngeal cancer and leukoplakia, and this method may contribute to reducing the need for biopsies [19].

Table 2 presents a summary of the conclusions from the studies discussed above.

**Figure 1:**
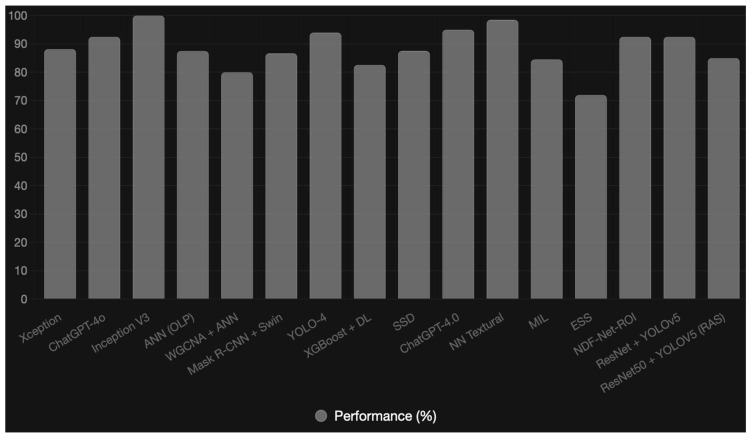
Comparative performance (%) of AI models from reviewed studies, based on the highest reported metrics (accuracy/AUC/F1-score normalized to % for comparability).

Figure 1 illustrates the comparative performance (%) of AI models from reviewed studies, based on the highest reported metrics (accuracy/AUC/F1-score, normalized to % for comparability). Data were aggregated from Table 1.

**Figure 2:**
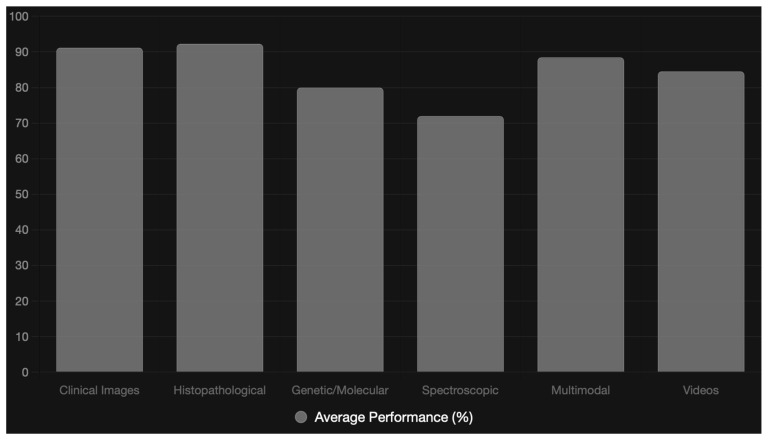
Comparison of data types by performance efficiency.

A comparison of data types by performance efficiency is shown in Figure 2.

**Figure 3:**
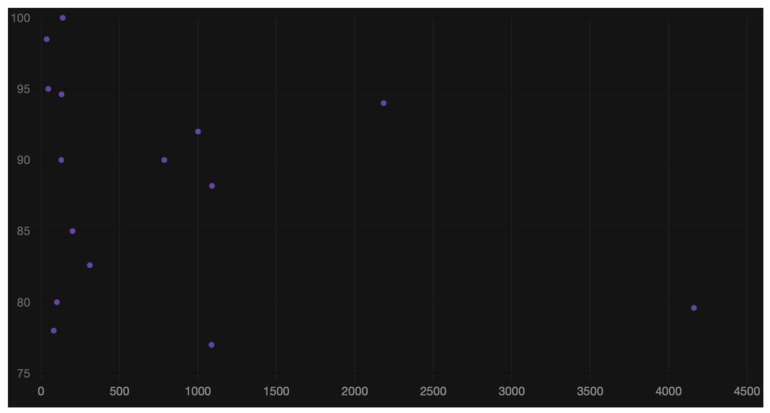
Relationship between accuracy and dataset size.

The relationship between accuracy and dataset size is illustrated in Figure 3.

**Figure 4:**
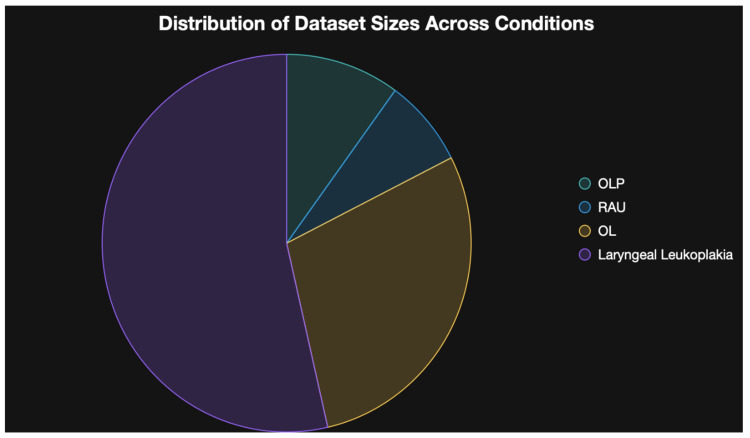
Distribution of dataset sizes across the conditions included in the review.

Figure 4 depicts the distribution of dataset sizes across conditions.

## 4. Discussion

Artificial intelligence methods, particularly machine learning and convolutional neural networks, show increasing potential in diagnosing dermatological and mucosal diseases.

In the presented publications on OLP diagnostics, predictive models, and clinical image analysis are most commonly used. Although AI models are mentioned in the context of image analysis, there is a lack of detailed references to specific algorithms and their implementation methods in clinical practice. It is worth noting that the reliability of studies using images of patients’ oral changes for artificial intelligence analysis may be limited by the quality and resolution of the photographs, which can vary depending on the technical conditions and equipment used. Although standardised photographic parameters were applied, this does not guarantee complete diagnostic reliability, as differences in lighting, capture angle, or camera settings may affect analysis results. The major difficulty in diagnosing OLP is its varied phenotype—not all forms of the disease are easy to unequivocally recognise. In the future, it would be beneficial to include a larger number of cases and additional variables, such as imaging data from ultrasonographic examinations. An alternative and potentially more reliable approach could be to utilize patients’ immunological biomarkers in conjunction with clinical images. In the context of the disease’s autoimmune basis, this approach could support the identification and prediction of OLP progression based on both clinical and laboratory data. The ultimate goal is to develop a model that integrates clinical, immunological, and imaging data to support the diagnosis of OLP in clinical settings. Such an approach could significantly enhance diagnostic effectiveness and enable more personalized therapy, especially in atypical cases. Additionally, greater emphasis is placed on models than on algorithms, suggesting the need to supplement studies with specific machine learning techniques, such as CNNs, which may be particularly useful in imaging diagnostics.

The success of ResNet50 and YOLOV5 models in classifying and detecting aphthous ulcers indicates significant potential for artificial intelligence models in future clinical applications for recognising oral mucosal abnormalities. Promising results presented in the study by Zhou et al. attest to the substantial possibilities offered by supporting the diagnostic process with computer analysis based on deep learning mechanisms [14]. Studies of this type form the foundation for developing more advanced and precise diagnostic tools based on deep learning mechanisms.

Currently, there are few studies on the diagnostics of oral leukoplakia using artificial intelligence, and the need for conducting such studies is increasingly emphasized by researchers. Work in this field is still at a preliminary and experimental stage. There is currently no programme that would significantly facilitate the diagnosis of this condition, replace traditional diagnostic methods, and improve detection at an early stage to prevent leukoplakia from transforming into cancer. Further research and AI model training on larger patient groups are necessary, considering a greater number of parameters and various lighting conditions. Particularly promising are studies that also include patient clinical history.

A recent study by Römer et al., part of the doctoral thesis of the University of Mainz’s co-author and student, Katharina Kloster, presents the first large-scale in vivo annotated dataset of the oral cavity using endoscopic hyperspectral imaging (HIS) under realistic clinical conditions. The authors demonstrated the feasibility of automated, non-invasive tissue classification across important anatomical intraoral structures. The large dataset provided a base for future work on pathological tissue detection, intraoperative margin assessment in oncology, and early, individualized diagnostics in oral medicine [8].

The use of artificial intelligence in diagnosing vocal fold and laryngeal pathological changes opens new perspectives toward faster, more effective, and more accessible pathology recognition. Although the presented studies show promising results in terms of AI system accuracy and efficiency, challenges remain related to limited dataset sizes, sample diversity. Undoubtedly, further code optimisation and adaptation to a broader database are necessary. There is, however, potential to utilize it in diagnostics via mobile devices, enabling physicians to remotely monitor patients’ conditions. A recent pilot study by Chau et al. aimed to evaluate the accuracy of GumAI, a mobile health tool based on artificial intelligence (AI) and smartphones, in detecting gingivitis. The study also examined user acceptance of personalized oral hygiene instructions. According to the authors, GumAI can potentially address gaps in dental care access, providing a cost-effective and accessible solution for the early detection of gingivitis and oral hygiene management [28].

However, it is essential to note that home-based self-assessment systems require special care and attention. There is a risk that the patient may overlook some oral lesions, for instance, due to difficulties in visualizing certain hard-to-reach areas of the mouth with the device. On one hand, positive feedback from participants supports the feasibility and acceptability of self-assessment tools, suggesting that with improvements, it could help address gaps in access to dental care [28]. On the other hand, technological optimism should be balanced with awareness of the risk of false negatives, especially in cases where lesions are problematic to access or visualize. False-negative results represent an especially important yet underreported limitation. Missed detection of lesions located in anatomically complex or difficult-to-access regions, such as the lateral tongue, floor of the mouth, or laryngeal structures, may delay diagnosis and negatively affect patient outcomes, particularly in potentially malignant disorders. Additionally, image acquisition quality plays a crucial role in AI performance; inadequate lighting, poor focus, and inconsistent imaging angles increase the risk of misclassification.

Despite these difficulties, further research and technological development suggest that AI will become an indispensable tool in laryngeal disease diagnostics, increasing access to medical care and improving patients’ quality of life.

By including laryngeal leukoplakia, this review illustrates that AI diagnostic models developed for oral mucosal diseases may also be applicable to other mucosal regions of the upper aerodigestive tract, emphasizing their broader practical relevance.

The variability in diagnostic performance reported across the reviewed studies reflects fundamental differences in model architecture, data quality, and validation strategies. Near-perfect accuracy was primarily observed in specialized CNN–based models evaluated under controlled experimental conditions, typically using standardized, high-quality clinical photographs and limited, well-curated datasets. While such results demonstrate strong technical feasibility, they may overestimate real-world performance and limit generalizability. In contrast, studies employing more heterogeneous clinical data or multimodal frameworks reported lower sensitivity, highlighting the challenges associated with anatomical variability, inconsistent image quality, and class imbalance in routine practice.

Histopathological confirmation remains a critical factor influencing diagnostic reliability. Studies incorporating biopsy-verified ground truth demonstrated more robust and clinically interpretable outcomes, whereas models trained exclusively on clinically annotated images were more susceptible to diagnostic ambiguity, particularly in conditions with heterogeneous phenotypes such as oral lichen planus. This distinction highlights the importance of interpreting high accuracy values with caution when histopathological validation is absent or limited.

Based on the current evidence, pre-trained CNN-based and hybrid models that integrate clinical, histopathological, or molecular data appear to be most effective. Nevertheless, existing AI systems should be regarded as supportive tools rather than independent diagnostic solutions, pending further large-scale and externally validated studies.

A significant limitation of the AI-based studies included in this review is the relatively small sample size, often derived from single-center datasets, which limits generalizability and increases the risk of overfitting. Therefore, despite high reported performance metrics, these results should be interpreted with caution.

Moreover, the included studies lack a unified and structured framework for evaluating AI models, with substantial differences in validation strategies and reported metrics. The absence of standardised assessment protocols and external validation represents an important limitation and a relevant point for further discussion.

Finally, most studies insufficiently addressed model uncertainty, confidence intervals, and the clinical impact of misclassified cases. Limited reporting on confidence estimation and class imbalance handling reduces transparency and hampers clinical applicability, particularly in the context of potentially malignant disorders.

## 5. Conclusions

Rapidly evolving artificial intelligence and deep learning algorithms enable increasingly accurate and faster detection of various diseases, including disorders of the oral mucosa. AI-based methods may become a valuable tool for physicians in the near future, supporting them in the diagnostic process. Applications that enable self-diagnosis at home may facilitate the earlier detection of potentially malignant changes and increase the likelihood of seeking a physician’s consultation. However, diagnostics using artificial intelligence methods currently require more precise studies and improvements.

## Figures and Tables

**Table 1:** The characteristics of the studies included in the review.

Authors	Year	Country	Disease/AI Application	Diagnosis	AI Study	Data	Image Acquisition	Conclusions
Achararit et al. [7]	2023	Thailand	OLP/non-OLP/diagnosis based on clinical images	Clinical + histopathological (100%)	Xception, ResNet152V2, EfficientNetB3	609 images of OLP and 480 images of non-OLP lesions	Clinical photographs obtained from the archive of the College of Dental Medicine, Rangsit University (8-bit JPEG); pixel values normalized to ensure numerical compatibility prior to CNN use	The best model was Xception, achieving an accuracy of 88.18% and an F1-score of 88.70%. The models were effective in diagnosing challenging cases
Yu et al. [9]	2024	China	OLP/diagnosis	Clinical only (diagnosis based on ICD-11 criteria and specialist evaluation; no histopathological confirmation reported)	CNN-based deep learning for automated recognition and diagnosis of OLP lesions ChatGPT-4O, Chat-Diagrams, Claude Opus	128 patients with OLP; images of mucosal lesions; divided into pre-trained and non-pre-trained groups	Clinical photographs of oral mucosa; images evaluated by specialists and analyzed by multimodal LLMs; technical preprocessing (resolution, normalization) not reported	Pre-training increases the effectiveness of AI in diagnosing OLP, including in rare locations
Osipowicz et al. [10]	2024	Poland	OLP/predictive analysis of clinical and histopathological data	Clinical diagnosis in 100% of cases; histopathological confirmation in 5.0% of cases	Artificial neural network (ANN)	80 patients suspected of having OLP; lesions were most frequently observed on the buccal mucosa, gums, and tongue	Clinical examination data (no image-based analysis)	The network achieved 71–85% accuracy, with the highest reliability for the buccal mucosa. Currently, the accuracy is insufficient for clinical practice. Larger datasets and integration of different types of data (clinical, imaging, immunological) are needed
Keser et al. [11]	2022	Turkey	OLP/identification	Clinical only (verified by oral medicine and maxillofacial radiology experts; no histopathology reported)	Google Inception V3 based on TensorFlow	65 images of healthy mucosa and 72 OLP cases	Standardized intraoral clinical photographs; images resized and divided into training and test sets; detailed preprocessing steps not specified	The AI model achieved 100% accuracy in classifying test images, correctly distinguishing between healthy and diseased mucosa
Idrees et al. [12]	2021	Australia	OLP/OLL/OED/via inflammatory cell analysis	Histopathological only	Artificial neural network (ANN)	Retrospective cohort of 130 cases with diagnosed OLP, OLLs, and OED with lichenoid host response	Digitized H&E-stained slides (40× scanner), stain normalization performed using QuPath “estimate stain vectors”; watershed-based nuclear segmentation applied	The applied AI model yielded encouraging performance, achieving AUC values of 0.982 and 0.988, with 100% sensitivity and 94.62% accuracy in distinguishing oral lichen planus from OLLs and OED with lichenoid host response
Qing et al. [13]	2023	China	OLP/molecular and clinical analysis with prediction of treatment outcomes	Not specified (likely histopathological for gene expression analysis, lesion count not applicable)	WGCNA (weighted gene co-expression network analysis) and Artificial neural networks (ANN)	Gene expression profiles clustered into five co-expression modules with 546 genes total	Gene expression profiling; preprocessing included normalization and clustering for WGCNA; image data not applicable	The model based on 100 differentiating genes achieved a predictive accuracy of 80%, surpassing traditional clinical diagnosis, which reached 60%
Zhou et al. [14]	2023	China	RAS/diagnosis	Clinical only (evaluated by two experienced physicians; percentage not specified)	Type of Artificial Neural Network—CNN (convolutional neural network) ResNet50 and YOLOV5	785 clinical oral photographs, which was divided into 251 images of RAU, 271 images of the normal oral mucosa and 263 images of other common oral mucosal disease	High-resolution oral clinical photographs; lesion boundaries manually annotated by experts; preprocessing included resizing and data augmentation (details not fully specified)	Pretrained ResNet50 and YOLOv5 models showed high performance in classifying and detecting RAU lesions on oral images, indicating that CNN-based AI has strong potential to support non-invasive clinical diagnosis in practice.
Cai et al. [4]	2025	China	OL/detection of genomic changes (loss of chromosome 9p) from histological samples	Histopathological only	Deep learning models (Twins-SVT, ResNet50, Inception_v3) and XGBoost for final classification	310 OL cases (217 training, 93 validation) and 365 HNSCC cases	Digitized histopathological whole-slide images; pathomics-based feature extraction; preprocessing adapted for deep learning and XGBoost classification	XGBoost achieved the highest performance for OL (AUC = 0.890 validation, 0.762 test). Promising for cheaper genomic analysis, but further validation on larger cohorts is needed. First application of deep learning to predict genomic alterations in head and neck squamous cell carcinoma (OLK and HNSCC).
Kouketsu et al. [15]	2024	Japan	OSCC/OL/diagnosis based on tongue images	Clinical diagnosis for OL; clinical + histopathological confirmation for OSCC	Single Shot Multibox Detector (SSD)	1043 images of lesions from 424 patients with OSCC (589), leukoplakia (49), and other oral mucosal diseases (405)	Standardized intraoral tongue photographs; Image acquisition was performed with fixed camera parameters, ensuring perpendicular alignment of the lens to the lesion surface. All photographs were acquired by trained oral surgeons and stored in JPEG format	Lower accuracy when including OL. Limitations: analysis limited to tongue surface and colour tone, poor lighting or small lesions caused false negatives.
Vinayahalingam et al. [5]	2024	Germany	Healthy mucosa/OL/OLP/diagnosis based on clinical images	Clinical only (ground truth based on expert annotation of clinical images)	Mask R-CNN (convolutional neural network) with Swin Transformer	4161 photographs (3337 training, 412 test, 412 healthy reference)	Standardized high-resolution clinical photographs; pixel-level manual annotation by specialists	Moderate performance for LP (F1 = 0.796, AUC = 0.938). LP diagnosis remains challenging; further improvements needed.
Kim et al. [6]	2023	Korea	Benign vocal fold tumors (cysts, granulomas, leukoplakia, nodules, polyps)/diagnosis based on endoscopic images	Clinical diagnosis (histopathology not specified)	Convolutional Neural Networks (CNN): Mask-50, Mask-101, YOLO-4, SSD-MN	2183 laryngoscopic images (349 healthy, 1834 with benign neoplastic lesions)	Endoscopic laryngoscopic imaging obtained from the hospital database; image quality suitable for remote and home-based screening	YOLO-4 achieved the best performance (F1 = 0.85, Acc. = 0.94). The system shows potential for home and remote diagnostics but needs more data for better accuracy.
Schmidl et al. [16]	2025	Germany	OSCC/OL/diagnosis based on clinical images and history	Clinical only (no histopathological confirmation included in AI assessment)	ChatGPT-4.0 (multimodal AI combining text and image analysis)	45 cases (including 15 OL)	Clinical photographs only, clinical data only, and combined; preprocessing for multimodal analysis was not fully detailed, although variations in data quality were noted to affect performance	With images only: OL Sens. = 72.2%, Spec. = 92.6%. With images + history: OL Sens. = 93.3%, Spec. = 96.7%. ChatGPT relied more on text than images; small sample size—further studies needed.
Jurczyszyn et al. [17]	2020	Poland, Germany	Mild leukoplakia (without high-grade dysplasia)/diagnosis based on clinical images	Clinical only (mild leukoplakia without histopathological grading)	Neural network using textural features (run-length matrix, co-occurrence matrix, Haar wavelet transform)	35 patients’ oral photographs	Standardized oral photographs (converted to 4-bit grayscale using dedicating software Labelme 4.5.6)	Sensitivity = 100%, Specificity = 97%. Promising results, but requires validation on a larger patient cohort.
Wang et al. [18]	2024	China	Benign and malignant vocal fold leukoplakia (VFL)/diagnosis based on using laryngoscopic videos	Clinical + histopathological (classification based on dysplasia severity, data size not specified)	Multi-Instance Learning (MIL)-based AI system	Laryngoscopic videos of patients divided into malignant (SCC, carcinoma in situ, severe dysplasia) and benign (inflammation, hyperplasia, mild dysplasia) groups	White-light laryngoscopic videos obtained via standard endoscopy	Accuracy = 85%, AUC = 0.851; higher than less experienced physicians (64.8%). AI support improved otolaryngologists’ diagnoses. Limitations: no normal VFL cases, only white light used.
Sakharkar et al. [19]	2025	USA	Laryngeal cancer/laryngeal leukoplakia/diagnosis based on elastic scattering spectroscopy (ESS)	Clinical diagnosis (reference diagnosis used for AI training; histopathology not specified for all cases)	Elastic Scattering Spectroscopy (ESS)	1086 ESS spectra from various oral sites; 20 participants; with cancer (10), leukoplakia (10)	Elastic scattering spectroscopy; obtained non-invasively from oral cavity sites	ESS technology combined with AI-assisted statistical modeling can accurately distinguish patients with laryngeal leukoplakia.

OLP—Oral Lichen Planus; non-OLP—diseases other than Oral Lichen Planus; OLL—Oral Lichenoid Lesions; OL—Oral Leukoplakia; OED—Oral Epithelial Dysplasia; OSCC—Oral Squamous Cell Carcinoma; SCC—Squamous Cell Carcinoma; RAS—Recurrent Aphthous Stomatitis.

**Table 2:** The summary of the conclusions in the presented studies.

Condition	Methods/AI Types	Performance Metrics	Dataset Size	Key Advantages	Key Limitations	Conclusions
Oral lichen planus (OLP)	Most frequently employed algorithms: Artificial Neural Networks (ANNs) and Convolutional Neural Network (CNN)-based models. Additional methods: Xception, ResNet152V2, EfficientNetB3, and Weighted Gene Co-expression Network Analysis (WGCNA) combined with ANNs.	Artificial Neural Networks (ANNs)—utilized in studies [10,12,13], demonstrating reliability with 71–85% accuracy [10], AUC values of 0.982–0.988 accompanied by 100% sensitivity and 94.62% accuracy [12], and 80% predictive accuracy [13]. Convolutional Neural Network (CNN)-based models in [9,11], achieving 100% accuracy in classification [11]. Additional methods: Xception/ResNet152V2/EfficientNetB3 [7], 88.18% accuracy, F1-score 88.70%); WGCNA + ANN [13].	Approximately ~1043 samples (609 OLP images [7] + 128 patients [9] + 4 confirmed cases [10] + 72 OLP cases [11] + approximately 43 OLP among 130 cases with mixed diagnoses [12] + 187 differentiating genes [13]. 813 confirmed OLP cases, 43 uncertain cases, 187 differentiating genes derived from an unknown number of OLP cases.	Clinical images—readily obtainable (via smartphone or camera), cost-effective, rapid, non-invasive, and facilitating remote diagnostics. Histopathological data—highly precise, validated through biopsy, suitable for inflammatory cell analysis. Genetic analyses—outperforming traditional clinical diagnosis (80% vs. 60%), providing a more economical alternative to comprehensive genomic sequencing.	Clinical images—image quality (e.g., lighting, resolution) impacts diagnostic reliability (potentially leading to errors in complex cases). Histopathological data—costly, invasive, and time-intensive (requiring laboratory processing). Genetic analyses—necessitating expensive assays and expanded datasets.	The highest-performing approaches: ANN with pre-training (enhancing efficacy in atypical lesion locations [9], and CNN Inception V3 (100% accuracy [11]. Overall reliability is high (mean > 80%), yet it is inadequate for routine clinical application without multimodal data integration [10]. Most advantageous: clinical images—yielding high accuracy (100% in [11]; histopathological data—(AUC > 0.98 in [12]. The implementation of AI is justified, as it outperforms conventional diagnostic approaches (e.g., 80% vs. 60% accuracy (WGCNA + ANN implemented in [13]; effective in challenging cases [7] and provides support for less experienced clinicians. However, at the current stage, human practitioners are superior in integrating multimodal data (clinical and immunological). AI does not yet demonstrate sufficient reliability for independent clinical use, requiring additional validation [10]. It shows promise as an auxiliary tool. Requirement for expanded investigations—the reviewed studies highlight the need for larger datasets to enable validation and data integration (e.g., [10] notes insufficient accuracy; [13] recommends larger cohorts to enhance reliability).
Oral Leukoplakia (OL) and Laryngeal Leukoplakia (LL)	Diverse: CNNs (Mask R-CNN + Swin Transformer, YOLO-4, MIL); ANNs/textural; Deep learning (ResNet + Transformer + YOLOv5, Twins-SVT/ResNet50/Inception_v3 + XGBoost); SSD; ChatGPT-4.0; ESS.	CNNs (Mask R-CNN + Swin Transformer [5], 79.6% F1 for LP; YOLO-4 [6], 85% F1, 94% acc.; MIL [18], 85% acc., AUC 0.851); ANN/textural features [16], 100% sens., 97% spec.; Deep learning (ResNet + Transformer + YOLOv5 [3], +2.46% acc. with ROI; Twins-SVT/ResNet50/Inception_v3 + XGBoost [4], AUC 0.890/0.762 for OL); SSD [4/22], lower acc. for OL; ChatGPT-4.0 [16], 93.3% sens., 96.7% spec. with history; ESS [18], AUC 0.77. Best performing: ANN textural (100% sens. [17]) and YOLO-4 (94% acc. [6]) for benign LP; XGBoost with deep learning for genomic (high AUC [4]). Overall reliability moderate-high (average 85–95%), but challenging for LP [5].	Approximately 3011 (unknown in [3]; 310 OL [4]; ~180 OL from 360 [4,15]; portion from 4161 for LP [5]; portion from 2183 for LP [6]; 15 OL [16]; 35 patients [17]; portion benign from videos [18]; 20 participants [19], only benign LL included, malignant excluded).	Photographs and endoscopic/laryngoscopic images and videos, and histological data Spectroscopy: non-invasive, promising for screening [19].	Spectroscopy: small sample size affects reliability, and lacks lifestyle factors.	Best performing: ANN textural (100% sens. [17]) and YOLO-4 (94% acc. [6]) for benign LP; XGBoost with deep learning for genomic (high AUC [4]). Overall reliability is moderate to high (average 85–95%), but challenging for LP [5]. Most beneficial: photographs/endoscopic—yielding high performance (94% acc. [6], 100% sens. [17]), easy to use; videos better for dynamic analysis (85% acc. [18]). Valuable, as AI improves diagnoses (higher than less experienced physicians, 85% vs. 64.8% [18]; promising for screening [19]); cheaper genomic alternative [4]. However, at this stage, humans are better equipped to handle complex cases (e.g., poor lighting/small lesions [4/22]); AI is not yet ready to handle cases independently (needs improvements [5], further studies [16]). Useful as support. More studies are needed—most articles indicate the need for further validation on larger cohorts [4], additional data [6], and consideration of small sample sizes [16,19] and uneven distribution [3].
Recurrent Aphthous Ulceration (RAU)	Primarily Convolutional Neural Networks (CNNs) (ResNet50 for classification, YOLOv5 for detection).	Exclusively clinical photographs (oral photographs) in [14]; literature analysis (images/results from databases) in [19]. No specific numerical results were given, although “high performance” was reported.	Approximately 251 (251 RAU images [14]).	Photographs (requiring validation on larger datasets [19]).	Lacking other methods, photographs predominate as straightforward and potentially clinically useful.	Reliability is high (“high performance” in classification/detection [14]. Pretrained ResNet50 + YOLOv5 performs best (strong potential in non-invasive diagnostics [14]. Average > 85% in similar frameworks from the review. Most beneficial: photographs—yielding high performance in detection and classification. AI is valuable as a support (strong potential in non-invasive diagnostics [14]; promising utility [27]. AI can accelerate diagnosis, but at this stage, it requires validation. Humans are better suited to handle complex cases, as AI is not yet ready for independent use. More studies are needed—both articles emphasize the importance of larger datasets for validation prior to clinical implementation [19].

## Data Availability

No new data were created or analyzed in this study. Data sharing is not applicable to this article.
